# China’s emergence and development challenges that China faces in Central Asia

**DOI:** 10.1007/s44216-022-00005-7

**Published:** 2022-12-28

**Authors:** Chi Zhang

**Affiliations:** School of International Relations, University of St Andrews, St Andrews, UK

**Keywords:** Central Asia, Sino-Russian relations, Terrorism, Belt and Road Initiative, Security cooperation

## Abstract

Development in Central Asia faces intensifying headwinds in various aspects. Terrorism and political instability have been the primary sources of concern for this chessboard of rivaling great powers. The US’s withdrawal from Afghanistan left a power vacuum, and the region’s future is further clouded by elevated uncertainty. The so-called ‘new Cold War’ discourse is becoming a self-fulfilling prophecy that contextualizes regional geopolitical maneuverings. These developments present a pressing need to evaluate development challenges in Central Asia in the context of China’s rising influence in the region through the Belt and Road Initiative and other regional frameworks, such as the Shanghai Cooperation Organization and China Pakistan Economic Corridor.

This paper seeks to examine the shifting geopolitical and geoeconomic landscape in Central Asia in the context of global ideological confrontations and the regional Great Game between China and Russia. Drawing on think tank reports, English-language media reports, and scholarly works, it argues that China’s investment and development strategy in Central Asia can be improved by giving geopolitical and geoeconomic factors full consideration. The changing political dynamics in the region have significant implications for China’s engagement with Central Asian countries, its broader Belt and Road Initiative extending through Central Asia to Europe, and development challenges that transcend the dualistic categorization of development and security.

## Introduction

Development in Central Asia faces intensifying headwinds in various aspects. Terrorism and political instability have been the primary sources of concern for this chessboard of rivaling great powers. The ongoing pandemic compounded transborder issues, as the lack of vaccines is also posing public health challenges to neighboring regions (Lehmann, 2021). Furthermore, the US’s withdrawal from Afghanistan left a power vacuum, and the region’s future is further clouded by elevated uncertainty. The so-called ‘new Cold War’ discourse is becoming a self-fulfilling prophecy that contextualizes the geopolitical maneuverings in the region. Speculation has spread internationally about China’s willingness and capability to further boost its presence in Central Asia’s development and security. These developments present a pressing need to evaluate development challenges in Central Asia in the context of China’s rising influence in the region.

Central Asia is strategically important to China for a few reasons. Economically, connected to the region through the Xinjiang Uyghur Autonomous Region (hereinafter Xinjiang), China has increased its investment in post-Soviet Central Asia more than 100-fold since 1991 (O’Reilly, 2015). Geographically, the borders that China shares with Kazakhstan, Kyrgyzstan and Tajikistan stretch over 3,300 km, offering great opportunity as well as nontraditional security challenges, such as terrorism, drug trafficking and illegal migration (Yuan, 2010). This resource-rich region is also a strategically important means for China to diversify its sources of energy supplies, which previously relied primarily on the Middle East and the Gulf (Yuan, 2010; Zogg, 2019).

Over the past decade, China’s ‘Going Out’ strategy has been further materialized by the Belt and Road Initiative (BRI), in which ‘Central Asia is an important and dangerous crossroads for China’s expanding global influence’ (O’Reilly, 2015). The BRI is not only a foreign policy project, and China’s investment in infrastructure abroad could also ‘help transportation of resources and goods between China and Central Asia, helping to develop China’s interior’ (O’Reilly, 2015).

China’s emergence in Central Asia has drastically changed the power dynamics in the region. Through the BRI, China will contribute to the $26 trillion needed for infrastructure investment in Asia until 2030 (Asian Development Bank, 2017), which could also help solve China’s problem of excess capacity (OECD Business and Finance Outlook 2018, 2018). However, China’s investment strategy in Central Asia has recently shifted from major infrastructure projects to manufacturing, and the diversification of economic portfolios fits with Central Asian countries’ expectations (Stronski and Ng, 2018: 11).

Against this backdrop, it is imperative to understand new challenges and opportunities arising from shifting political and economic dynamics in the region. This paper seeks to examine Central Asia’s shifting geopolitical landscape in the context of global ideological confrontations and the regional Great Game between Russia and China. Development challenges will be analyzed in the context of China’s engagement with Central Asia. This focus limits the scope of this paper to four topics – Sino-Russian competition, which sets the scene for the rivalry between these great powers; Central Asia’s reception of China’s influence, which offers a less-explored but much-needed perspective on China’s expanding power; instability in Afghanistan after the US’s withdrawal, which addresses the most recent major change in Central Asia’s geopolitical landscape; and the threat of terrorism, which is often considered China’s greatest concern without further scrutiny.

By tracing the recent developments in the region, this paper qualitatively examines the challenges for development in Central Asia. The data, collected from think tank reports, English-language media reports, and scholarly works, are analyzed through a geoeconomic framework, which facilitates a better understanding of the relationship between the wider geopolitical dynamics and China’s ‘geostrategic use of economic power’ (Wigell, 2016: 137) among BRI countries in Central Asia. By doing so, this paper seeks to identify risk factors arising from the shifting great power rivalry and local conditions in the region.

The changing political dynamics in the region have significant implications for China’s future engagement with Central Asian countries, its broader BRI extending through Central Asia to Europe, and development challenges that transcend the dualistic categorization of development and security (Allouche and Lind, 2013).

The remainder of this paper will proceed as follows. The second section will contextualize the discussion with a brief overview of Sino-Russian relations, focusing on the countries’ shared interests, competition, and friction in various realms. The third section will complement the Russia-centered discussion by focusing on Central Asian states’ reception of China’s expanding influence in the region. The fourth section will focus on the instability in Afghanistan after the US’s chaotic withdrawal in 2021 and its implications for regional security. The fifth section will explore the threats posed by terrorism and extremism in the region.

## Sino-Russian competition

This section provides a brief overview of the regional Great Game between Russia and China to contextualize the discussion of China’s emergence in Central Asia. Given the drastic change in the geopolitical landscape, the roles of the two great powers cannot be underestimated. Their relations are key to stability in Central Asia. Confronted with talk of a ‘new Cold War’, Russia and China have been pushed into each other’s arms as the US considers both as sources of security threats, and thus, they have more shared interests than potential areas of conflict when the US remains the top rival for both countries.

Russia and China have the same concerns about ‘color revolutions’ perpetuated by the US-led international system (Stronski and Ng, 2018; Yuan, 2010), which have overthrown a number of post-Soviet governments (Piekos and Economy, 2015). In particular, the ‘color revolutions’ in Ukraine and Kyrgyzstan and the 2005 Andijan incident in Uzbekistan pushed Russia further into the arms of the SCO and prompted it to engage more actively in counterterrorism cooperation in the region (Yuan, 2010). In other words, both countries are concerned about ‘any American strategic projection into resource-rich areas on their borders’ (O’Reilly, 2015). As the US ‘became more engaged actively in East Asia, and began lobbying for NATO expansions into Georgia and Ukraine’, Russia and China became more strategically aligned (Asiryan and He, 2020). This tendency has become clearer since Russia’s invasion of Ukraine in February 2022.

Despite the shared interests, observers often view Sino-Russian relations as temporary and fragile – a so-called ‘axis of convenience’ (Lo, 2008). Indeed, as Sino-Russian relations are driven more by interests rather than identity, the withdrawal of the US from Afghanistan represents a significant shift in the political dynamics in the region.

In this context, Russia has not been very enthusiastic about China’s presence in the region. First, while the Shanghai Cooperation Organization (SCO) is led by both China and Russia, China has been active in carrying out military exercises within this framework, making it a potential competitor of the Russia-led Collective Security Treaty Organization (CSTO). Nevertheless, although incorporating ‘all of the CSTO’s Central Asian members plus Russia, China, India and Pakistan’, SCO does not challenge Russia’s dominance in terms of *military defense* in the region (Weitz, 2018: 74).

There are some concerns over an alleged military base in Tajikistan funded and built by China (Eurasianet, 2020; Y Jiang, 2021a, b; Zogg, 2019). It is said that the base, agreed upon between Tajikistan’s Interior Ministry and China’s Public Security Ministry, with $10 million from China, will focus on ‘counterterrorism amid arising concerns over instability in neighboring Afghanistan’ (Devonshire-Ellis, 2021). However, such reports have been dismissed by both China and Tajikistan (ANI, 2021).

China’s defense policy provides that China will not establish any military base abroad, and the one in Djibouti is framed as a ‘logistics’ base. Therefore, although there may be military cooperation between China and Tajikistan, it is likely to be conducted within the existing regional frameworks. Publicly establishing a military base would be an open violation of China’s own defense policy, which would be a sign of a major shift in China’s approach to and intended level of influence in Central Asia. Nevertheless, this example indicates that although China is constrained by its noninterventionism in regard to regional military cooperation, concerns exist in the region regarding its deepening military engagement. China’s limited scope for military cooperation with Central Asia is not considered a threat to Russia, which has a close working relationship with Tajikistan within the CSTO (Umarov, 2021). Furthermore, Russia does not feel threatened, as Beijing does not seek to diminish Russian influence – Beijing's military activities in the region are directed at securing its own security interests (Umarov, 2021).

Second, power asymmetry in the region is exacerbated by economic asymmetry. China has become increasingly influential in Central Asia, with its economy eight times the size of Russia’s (Zogg, 2019). ‘As the SCO has achieved significant results in its campaign to crush terrorists’ movements in Central Asia’, according to Wang Yiwei, director of the Institute of International Affairs at Renmin University, ‘economic cooperation has inevitably been put on the agenda of the organization’ (Zhang and Yang, 2021). The SCO’s role in facilitating economic development is corroborated by its Secretary-General, Vladimir Imamovich Norov: ‘investment cooperation has become a significant agenda item within the SCO’ (Zhang and Yang, 2021). The Russia-led Eurasian Economic Union (EAEU) has been overshadowed by China’s BRI (Stronski and Ng, 2018; Weitz, 2018; Zogg, 2019). The Greater Eurasian Partnership, proposed by Putin in 2016, seeks to deepen Russia’s economic partnership across Eurasia and further consolidate Russia’s reach in the far east (Sahakyan, 2021). Interviews with Russian and Kazakh elites indicate skepticism toward Russia’s ‘Greater Eurasian Partnership’ and more ‘divergent opinions’, including positive views of and support for the BRI (Shakhanova and Garlick, 2020: 49).

As China’s investment soars in Central Asia, power asymmetry between Russia and China is likely to feed the former’s insecurity toward the latter (Rolland, 2019: 15). China is a major practitioner of geoeconomics, and its economic power has given it leverage in inducing desired behavior from Central Asian countries. Holding the ‘upper hand in the relationship’, China now has the capability to ‘grow at the expense of Russia’, although it may decide not to do so (Stronski and Ng, 2018). This power asymmetry is likely to continue, if not be made worse, by sanctions placed on Russia for its war against Ukraine, although China’s growth is also slowed by its zero-COVID policies.

This shifting power asymmetry poses a challenge no less than the US’ geopolitical maneuvering in the region. As Alexander Lukin (2021: 170) notes, ‘[a]ny possible changes in US policy will probably prove less of a deterrent to further Russian-Chinese rapprochement than will Russian concerns over China’s growing assertiveness’.

As American influence dwindles in the region, the geopolitical rivalry between the two powers is potentially destabilizing. If they decide to ‘back different factions to project their regional power’, this will create opportunities for terrorists to spread extremist ideology and violence in the common backyard of Russia and China (O’Reilly, 2015).

Furthermore, the divergence of Chinese and Russian interests, especially regarding energy and economic integration in Central Asia, could manifest in incidents such as the Georgian crisis (Turner, 2011; Wishnick, 2009). Russia’s pursuit of power is considered at odds with China’s expanding influence in the energy arena (Freeman, 2018). ‘In 2009, the completion of pipelines to China broke the Russian monopoly on energy outlets for the region’ (Zogg, 2019).

While China’s interest-based ‘new regionalism’ has limitations, as discussed above, it is more flexible than the traditional identity-based regionalism, exemplified by the EU (Chung, 2004: 993). This means that linguistic and cultural barriers are less of a problem for the tripartite relationship among China, Russia and Central Asia than they would have been in the EU.

While examining key players, Russia and China, and the Great Game narrative is helpful in sketching out the ‘big picture’, it is insufficient to capture the complexity of the geopolitical landscape because it gives little agency to the Central Asian states (Asiryan and He, 2020). To complement the above analysis, the following section will focus on Central Asia’s reception of China’s expanding influence in the region.

## Central Asia’s reception of China’s influence

This section focuses on how Central Asian countries have responded to Russian and Chinese influence. The agency of Central Asian countries has been overshadowed by discussions of rivalries between major powers. The attitudes of Central Asian countries are important for China to fine-tune its development strategy in the region.

China’s expanding influence in the region is gradually changing locals’ perception of China. Unfavorable views of China have long existed in the region. Before the 1980s, the Soviet propaganda apparatus spread ‘extremely negative clichés about China’ (Peyrouse, 2016: 14). Since the outbreak of COVID-19, conspiracy theories regarding the origin of the virus have exacerbated anti-China racism across the globe. Already a geopolitical battlefield, postpandemic Central Asia has become an arena for further Sino-Russian rivalry in terms of ‘vaccine diplomacy’ (Putz, 2021).

While Central Asian countries enjoy close ties with Russia as former Soviet republics, they have also countered Russia’s aggressive foreign policy through regional organizations and platforms that help diversify their ties to contain Russia’s rising influence in the region (Tskhay and Costa Buranelli, 2020: 1034). With China’s increasingly dominant presence in Central Asia, these countries will continue using regional frameworks for hedging purposes to balance China’s emergence.

The current global anti-China sentiment poses further challenges to China establishing itself as a responsible regional power in Central Asia. China’s growing economic power and global influence can be viewed as threats when a record high number of people hold unfavorable views of the country (Silver et al., 2020). Although Central Asian governments generally maintain good relations with the Chinese government, public opinion places constraints on these governments. For example, Kazakhstan saw a series of protests against a planned land reform in 2016 and Sinophobic protests against Chinese companies (Reuters, 2019). Similarly, survey data from the Central Asia Barometer indicate increasing opposition in Uzbekistan to Chinese energy and infrastructure development in the region (Trilling, 2020).

Furthermore, although the myth of ‘debt trap diplomacy’ has been debunked (Lee and Shahar, 2020), fear remains that the BRI’s lending mechanism will increase Tajikistan’s risk of debt distress (Chen, 2018) and debt dependence more generally in Central Asia (Tsikhay, 2021). The economic reliance developed along with the BRI does not help the countries deal with the structural problems facing their economies. Debt dependency is a concern not only for receiving states but also for China. The COVID-19 pandemic has put pressure on Central Asian countries, which already have low sovereign credit ratings, to pay off Chinese debts (Honig, 2020).

Another factor that may impact Central Asia’s reception of China is the Russian media. Considering the impact of Russian media on Central Asia, their attitudes toward China also present a challenge to China’s image in the region. Kumakura (2021: 305) notes a sign of change in the Russian media, which have started swaying public opinion on China’s presence in Central Asia. This represents the shift of attitudes within Russia, where as Peyrouse observed, Sinophile circles were forming (Peyrouse, 2016: 18).

China’s growing economic power in the region has increased demand to learn the Chinese language. The trend is seen in both schools and private education companies. For example, Chinese is becoming more popular than Russian in Tajik schools (Altynbayev, 2021). Nurzhan Baitemirov, founder of East‒West Education Group, is shifting his focus from English to Chinese (Farchy, 2016). Under Xi, China has invested heavily, within the SCO framework, in education diplomacy. Early in 2013, Xi visited Nazarbayev University, offering 30,000 scholarships and inviting 10,000 teachers and students of the Confucius Institute to visit China (Xi, 2013). Since then, there has been a steady increase in the numbers of students from Central Asian countries receiving Chinese government scholarships (JY Jiang, 2021a, b). There have been some successes in cultivating positive views toward China among Central Asian students (Chen and Jiménez-Tovar, 2017; Yau, 2021).

The efforts to promote education diplomacy notwithstanding, Chinese language learning presents formidable challenges, as it is from a different language family than the Turkic and Slavic language families dominant in the region, and many students only complete the beginner level (Kumakura, 2021: 304). This is compounded by a perceived ‘civilizational difference’ and ‘impassable cultural barrier’ between Central Asia and China (Peyrouse, 2016: 22).

While China’s ‘new regionalism’ in Central Asia is interest-based, identity factors still play a role. For example, the friendship sustained by investment was tainted by demonstrators at the Chinese consulate in Almaty, who demanded the release of their relatives allegedly detained in Xinjiang (Rymbetov, 2021). Incidents such as this have compounded the existing animosity against China’s growing influence in the field of oil industry (Rymbetov, 2021).

Given China’s interest-based engagement with Central Asia, identity-related risks do not pose an immediate challenge to China’s geoeconomic strategy in the region. To date, all the Central Asian countries have supported China’s ethnic policy in Xinjiang. However, in the long run, identity-led distrust may limit China’s influence in economic cooperation. It remains a potential factor that may prevent China from translating its economic power into actual leverage on controversial issues such as its approach to the Uyghur population in Central Asian countries.

## Instability in Afghanistan and its spillover in Central Asia

This section focuses on recent developments in Afghanistan. The situation in Afghanistan is particularly important after the US withdrew its military presence on 30 August 2021, leaving the Taliban to regain control. As this section will show, the return of the Taliban is a significant factor for Central Asia’s wider geopolitical landscape.

Central Asia has already been considered a ‘hotbed for extremism’ due to its economic hardship and repression (Dalton, 2017), although the pervasive view that it poses a danger to the West is largely unfounded (Heathershaw and Megoran, 2011). The ‘discourse of danger’ related to Central Asia contributes to the securitization of the region and justification of the US’s war in Afghanistan (Cooley, 2021: 1991–2021). However, regardless of the extent of securitization before the US’s withdrawal, the Taliban’s takeover of Afghanistan resulted in exacerbated chaos, leading to the spillover of the humanitarian crisis, drug economy and terrorism risks.

The withdrawal of US troops marked the end of 20 years of US war, which cost thousands of lives and trillions of dollars (Associated Press, 2021). As one of the poorest countries and the largest illicit opiate supplier, Afghanistan was already suffering from a grave humanitarian crisis before the situation was aggravated by COVID-19. ‘In 2019 there were more than five million Afghan asylum seekers, refugees and internally displaced persons’ (Select Committee on International Relations and Defence, 2021: 4), presenting a grave challenge to the country’s immediate neighbors.

Externally, Afghanistan relies heavily on foreign aid, and internally, it relies heavily on opium production. In 2017, opium production was estimated to be ‘worth some $1.4 billion in sales by farmers or roughly 7% of Afghanistan’s GDP’, according to the UN Office of Drugs and Crime (Landay, 2021). As many rural jobs are dependent on poppy cultivation, there are concerns, for example, within the UK (Select Committee on International Relations and Defence, 2021: 5), that humanitarian assistance could unwittingly sustain the narcotics trade.

Political instability has also increased the risk of investing in Afghanistan’s natural resources, which are estimated to be worth approximately $1 to $3 trillion (Azad, 2020; Reuters, 2021a). There have been a few Chinese attempts at investing in natural resources. Jiangxi Copper Co Ltd. and Metallurgical Corp of China took over the Mes Aynak copper mine in 2008, but construction has been delayed by instability (Zhang and Singh, 2021). In 2011, the former cabinet of Afghanistan approved a deal to allow the China National Petroleum Corporation to carry out oil exploration and extraction in the Amu Darya Basin over the following 25 years (BBC, 2011). This project was halted by local militia (Xin Kuaibao, 2012) and disagreements over the transportation of oil out of Afghanistan (Tolonews, 2013). These two examples capture the difficulties in investment in Afghanistan’s natural resources.

The chaos in Afghanistan has already spilled over into Pakistan. The unguarded 2,570-km border (Landale, 2021) and political and ideological linkages between the two countries make it difficult to separate Islamic militancy between them (Mufti, 2012). Terrorism, on the rise since 2017, culminated in August 2021 with 35 attacks that killed 52 (Mangi et al., 2021). Emboldened by these developments in Afghanistan, militant groups in Pakistan have already been keeping investment away (Mangi et al., 2021). Attacks by the Tehrik-i-Taliban Pakistan (TTP) surged with the Afghan Taliban’s takeover (Grossman, 2021). The TTP has merged ten militant groups since July 2020, including ‘three Pakistani affiliates of al-Qaeda’ (Sayed, 2021). Upon its takeover, the Afghan Taliban released hundreds of TTP members to pave the way for full ceasefire negotiations (Reuters, 2021b).

The renewed security challenges posed by the developments in Afghanistan have increased Central Asian countries’ reliance on regional organizations such as the CSTO and SCO (Mallinson, 2021). As the SCO expanded to include India and Pakistan in 2017, it has given Russia and China greater leverage to address instability in Afghanistan. The inclusion of these two countries will help China and Russia develop multilateral platforms that are conducive to a more multipolar world order to counterbalance the US (Stronski and Ng, 2018: 15).

In this context, postconflict reconstruction is the precondition for any major investments. China stepped in almost immediately after the US’s withdrawal to provide relief, announcing $31 million worth of emergency aid, including food and water supplies, COVID-19 vaccines and medicine, on 8 September 2021 (CGTN, 2021). A week later, on 13 September, the US announced nearly $64 million of humanitarian assistance (USAID, 2021). The West’s reluctance to provide funds may drive Afghans closer to China and Pakistan – two countries that responded quickly to its humanitarian crisis (Greenfield, 2021a).

In addition to government-to-government aid, China’s involvement in postconflict reconstruction in Afghanistan is likely to be rolled out through regional frameworks. At a meeting of the heads of the SCO, President Xi emphasized the SCO’s role in facilitating a smooth Afghan transition and developing an inclusive political structure (Al Jazeera, 2021).

Discussions were underway, even before the US withdrawal (Xie and Chu, 2021), to include Afghanistan in the China-Pakistan Economic Corridor (CPEC), under which China has pledged over $60 million for infrastructure projects (Greenfield, 2021b). The extension of the CPEC to Afghanistan was endorsed by Hua Chunying, a spokesperson for the Ministry of Foreign Affairs: ‘China supports the CPEC’s extension to Afghanistan so that the Afghan people can benefit from the BRI’ (Rehman, 2020). Some scholars have expressed similar views (Xie and Chu, 2021).

Among other postconflict reconstruction tasks, a key challenge to any further development in Afghanistan is the condition of its roads. In the past two decades, the country’s 2,000-mile Ring Road has been repeatedly rebuilt and destroyed by terrorist attacks. The importance of road infrastructure to Afghanistan’s development and governance is captured in a Special Inspector General for Afghanistan Reconstruction (SIGAR) report: ‘if the Kabul to Kandahar highway were to become impassable, the central government would collapse’ (Special Inspector General for Afghanistan Reconstruction, 2016b: 39). With $2.8 billion spent by 2016 (Special Inspector General for Afghanistan Reconstruction, 2016a), Afghanistan’s road infrastructure was crumbling when the Taliban took over (DW, 2021).

The US’s approach to building the Ring Road was one of nation building. The Ring Road is designed to facilitate the delivery of public services and humanitarian aid; thus, it was expected to bring legitimacy to the central government and to enable the US and NATO to send troops and supplies to keep the Taliban in check (Ellis, 2018). However, as the US’s attention was diverted to Iraq after 2003, it cut its funding for road construction in Afghanistan. WikiLeaks geospatial attack data show that Taliban attacks escalated from 2004 to 2009 and were concentrated along the Ring Road (Shachtman, 2018). The absence of major roads that connect within Afghanistan prevents the transportation of supplies for any other development projects.

While the US’s ‘nation-building’ approach did not succeed, road construction must be carried out alongside nation-building efforts. Research on the relationship between opium and road construction demonstrates that ‘where the rule of law is limited and opportunities for legal livelihoods remain scarce, investments in physical infrastructure can inadvertently incentivize illegal economic activity’ (Wigton-Jones, 2021: 405).

Afghanistan was already subject to multiple sanctions when the Taliban took over, and the country’s economy was suffering from a severe drought, armed conflict and COVID-19. ‘[I]ts healthcare systems, and wider social fabric are heavily dependent on foreign assistance’ (Moret, 2021). A recent report highlights the unprecedented food crisis in Afghanistan, where ‘nearly 19 million people are highly food insecure due to prolonged drought, conflict and economic collapse’ (Integrated Food Security Phase Classification, 2021: 1).

With international sanctions in place, the private and not-for-profit sectors may face serious constraints in supply chains and financial exclusion – a phenomenon known as ‘overcompliance’, ‘derisking’ and the ‘chilling effect’ (Moret, 2021). This may exacerbate the existing humanitarian crisis in Afghanistan.

As calls for ‘strategic relief of some existing US and UN sanctions’ (Moret, 2021) are being made, there is a window of opportunity for China to engage in political dialog with the Taliban. Indeed, China called for lifting economic sanctions on Afghanistan, which would contribute to its reconstruction (Rachel, 2021). Although the US compromised by proposing humanitarian exceptions to the existing sanction regimes, it remains challenging to reach international consensus on the provision of humanitarian assistance under the 1988 Afghanistan sanctions regime (United Nations Security Council, 2021a). Opponents of the US’s initial proposal of 9 months, such as China and Russia, argued that the exemption that would allow humanitarian assistance should not be subject to a time limit but were willing to accept a 12-month period. Others, including France, Estonia, India and the UK, advocated for a shorter time limit of 6 months (United Nations Security Council, 2021a).

## Terrorist threats

The issue of terrorism captures the intertwined nature of security and development, as the ‘root causes’ of terrorism are often considered embedded in the socioeconomic conditions of certain disadvantaged groups or individuals. Although observers often cite the issue of East Turkistan forces as China’s major concern in the region, this issue is conflated with the controversies surrounding deradicalization programs in Xinjiang and is understood, especially in Western media, as an excuse for power abuse. The conflation of these two issues obscures the actual scale of Uyghur militancy. This section seeks to examine terrorist activities that may cause significant disruption to development by actors, including the terrorists who target Chinese interests and other local terrorist networks that pose a threat to economic development more generally. For the purpose of this paper, ‘Uyghur militants’ are defined as Uyghur individuals who advocate for an independent ‘East Turkistan’ (what the Chinese authorities call the Xinjiang Uyghur Autonomous Region) *through violent means*. While the designation of terrorists according to China’s Anti-Terror Law remains controversial, it is beyond the scope of this paper and discussed elsewhere (see Zhang, 2019). This paper does not assume that Uyghurs, predominantly Sunni Muslims, are more susceptible to radicalization than any other groups. Given that ethnic relations between Uyghurs and Han have suffered from tensions intensified in the Sino-US rivalry, a small number of radicalized Uyghurs found their way to radicalism, joining forces with extremists in Central Asia and the Middle East. Uyghur militants have operated under several names but have been given the label of ‘East Turkistan forces’ by the Chinese authorities.

The labeling of all the Uyghur activist groups and individuals as ‘East Turkestan forces’ is misleading for three reasons. First, it obscures the dynamics within the group. Under the mistranslated East Turkistan Islamic Movement (ETIM), the Turkic Islamic Party (TIP) can be viewed as a jihadist wing, and the Istanbul-based, Uyghur-led East Turkistan Education and Solidarity Association (ETESA) can be viewed as an Islamic wing (Zenn, 2018). The latter is not considered a terrorist organization in most countries, which poses challenges to China, making it difficult to facilitate international counterterrorism cooperation.

Second, lumping Uyghur terrorist groups together as ‘East Turkestan forces’ does not help distinguish terrorists from the law-abiding Uyghur diaspora whose support is crucial to the legitimacy of China’s deradicalization programs. According to a UN report, several hundred members of the TIP are located in Badakhshan and Afghanistan (United Nations Security Council, 2021b: 19). This small group of militants must be differentiated from the 10 million Uyghurs who currently reside in Central Asia (IRIN News, 2001). According to Salih Hudayar, the Uyghur president and founder of the Washington-based East Turkistan National Awakening Movement, although Uyghurs in China and the people of Afghanistan belong to the Hanafi sect of Sunni Muslims, most Uyghurs are liberal and view the Taliban as very extreme (Kashgarian, 2021). It remains difficult for the Chinese authorities to differentiate between violent extremists and local supporters in Central Asia who have family in Xinjiang. Although Central Asian governments are generally cooperative within the SCO framework, it is in the interests of the Chinese government not to alienate the 10 million Uyghurs in Central Asia.

Third, terrorists from Xinjiang have been working with local terrorist organizations. Given the support from local terrorist networks to help a small number of Uyghurs flee to Syria, assessing the connections between terrorists from Xinjiang and terrorist networks in Central Asia is a prerequisite for any development plans in the region. In 2008, the TIP released its first video indicating an ideological affinity with al-Qaeda and loyalty to the Taliban (Zenn, 2018). In the Syrian civil war, some have shifted loyalty to the self-proclaimed Islamic State in Syria (Zenn, 2017). In 2016, when the Syria Civil War was at its height, terrorists from Xinjiang heightened their presence with a few propaganda videos explicitly targeting China, some of which included scenes of child soldiers. Uyghur participants in the Syrian Civil War operated as part of the TIP, also known as ‘Katibat Turkistani’, translated as the ‘Turkistan Brigade’ (Clarke and Kan, 2017).

The exact number of Uyghur foreign fighters has been subject to debate, ranging from 300, as estimated by the Chinese authorities, to 5,000, a number given by Syria’s ambassador to China (Blanchard, 2017). The actual number of Uyghur foreign fighters likely rose from a few hundred in 2013 to over 3,000 in 2020, based on various sources (Botobekov, 2017; Israeli Intelligence Heritage and Commemoration Center, 2014; Rosenblatt, 2016; United Nations Security Council, 2020; Watts, 2016).

As China has become a prominent target for terrorists from Xinjiang, the instability in Central Asia provides an arena for scattered terrorists to regroup and renew their connections with local terrorist networks such as the Hayyat Tahrir al-Sham (HTS), Hizb ut-Tahrir (HuT), Islamic State—Wilayat Khorasan (ISWK) and local Turkestan Islamic Party (TIP) (The SecDev Group, 2018). Attacks against Chinese interests in the region, such as the 2016 Chinese embassy bombing in Bishkek, are likely to reoccur (Zenn, 2017).

Adding to the difficulty of identifying terrorists, it remains unknown how many foreign terrorist fighters from Central Asian countries were Uyghurs. Central Asian countries have been recognized as one of the major sources of foreign terrorist fighters who travel to conflict zones to engage in terrorist activities. The number of foreign fighters from Central Asia to Syria was between 2,000 and 5,000 (Lemon, 2018). The number of foreign terrorist fighters from areas including Central Asia, Russia, Pakistan and Xinjiang was between 8,000 and 10,000 in June 2020 (United Nations Security Council, 2021b: 18).

The situation has been further complicated by the US’s withdrawal from Syria and COVID-19. The US pullout was accompanied by reports of prison breaks of hundreds of ISIL prisoners (Al Jazeera, 2019). More prison breaks were reported in March as global attention shifted to COVID-19 (McKernan, 2020). It remains unknown whether any terrorists from Xinjiang were among these prisoners.

China’s high level of control over its borders has thus far helped keep terrorists away from its territory. However, Central Asian countries remain strategically important for China’s domestic security because they are likely to be chosen as a haven and a staging ground to launch further attacks against China (Clarke and Kan, 2017: 11). Some returning fighters have already been reported in Afghanistan (Small, 2017).

Online violent extremist content permeates Central Asian social media. A 2018 report ‘found 140 Central Asian social media accounts that were actively distributing VE [violent extremism] content to over 324,000 subscribers’ even though content-blocking mechanisms were in place; more effective means such as education and engagement were underdeveloped in the region (The SecDev Group, 2018: 3). As long as China continues to ‘go out’, stability in Central Asia remains a key factor in protecting Chinese overseas interests.

On the other hand, Central Asia provides a key multilateral platform for China to carry out counterterrorism activities, even though Afghanistan is not a member of the SCO. Central Asia plays a facilitating role in Afghanistan’s peace and security process (Guterres, 2018). Tajikistan shares a border with Afghanistan that is approximately 1,300 km long, allowing for terrorist infiltration (Weitz, 2018: 8). The ‘Quadrilateral Cooperation and Coordination Mechanism in Counter-Terrorism’ was established in 2016 by Afghanistan, China, Pakistan and Tajikistan to facilitate multilateral regional security coordination beyond the SCO (Korybko, 2019). In August 2020, China convinced Pakistan to open five key border crossings to allow bilateral trade with Afghanistan and transit trade of Afghan exports to India (South Asia Monitor, 2020), which also decreased ‘Afghan’s dependence on the Chabahar port in Iran’ (Azad, 2020). Even China’s military activity ‘near the Tajik-Afghan border has more to do with Afghanistan than Central Asia’ (Asiryan and He, 2020).

## Conclusion

This paper has evaluated the threat posed by geopolitical rivalry, the geoeconomic landscape, regional instability, and terrorism in Central Asia. These security challenges have great implications and policy relevance for development and for China’s involvement in this region. The discussion of the four interconnected topics has shown that to understand China’s emergence and the development challenges that it faces in the region, China’s engagement with Central Asian countries cannot be severed from the geopolitical and geoeconomic landscapes and the existing security threats that already confront the region. This paper concludes with the following four observations.

First, constructive Sino-Russian relations that focus on shared interests are conducive to regional security. The regional frameworks led by the two countries may become competitive, for example, CSTO versus SCO and EAEU versus BRI. However, it is in the interests of regional peace that the two countries develop their respective competitive advantages to develop the region together. If the two engage in zero-sum competition, the region will rapidly descend into a battlefield of proxy wars and a source of terrorism and refugees.

Second, while China’s education diplomacy is cultivating positive opinions toward China in Central Asia, more needs to be done to address postpandemic anti-China sentiments that are rooted in the Soviet era. Having been influenced by the Russian language and culture for decades, people in Central Asia need time to catch up with the shifting power dynamics.

Third, Afghanistan, which has recently seen the departure of the US, presents considerable challenges to regional security. The Taliban’s former ties to international terrorist organizations cannot be severed completely, as exemplified by the release of TPP prisoners. Its economy, which is highly dependent on aid and opium production, is in dire need of structural reform before humanitarian aid can truly benefit the people. Infrastructure projects such as roads are crucial to pave the way for peace in landlocked and mountainous countries such as Afghanistan. At the same time, building infrastructure without nation building could inadvertently contribute to the illicit drug trade.

Fourth, to create conditions for better counterterrorism cooperation, efforts must be made to differentiate terrorists and the 10 million Uyghur people residing in Central Asia. Further research needs to evaluate the *actual* capability of Uyghur militants to launch any attacks against China. Such research will also help narrow the overbroad counterterrorism agenda, a subject of much international criticism.

## Data Availability

Not applicable.
